# Stability of SG1 nitroxide towards unprotected sugar and lithium salts: a preamble to cellulose modification by nitroxide-mediated graft polymerization

**DOI:** 10.3762/bjoc.9.181

**Published:** 2013-08-06

**Authors:** Guillaume Moreira, Laurence Charles, Mohamed Major, Florence Vacandio, Yohann Guillaneuf, Catherine Lefay, Didier Gigmes

**Affiliations:** 1 Aix-Marseille Université, CNRS, ICR UMR 7273, 13397 Marseille, France; 2 Aix-Marseille Université, CNRS, MADIREL UMR 7246, 13397 Marseille, France

**Keywords:** ESR, glucose, lithium salt, nitroxide, SG1, stability

## Abstract

The range of applications of cellulose, a glucose-based polysaccharide, is limited by its inherently poor mechanical properties. The grafting of synthetic polymer chains by, for example, a “grafting from” process may provide the means to broaden the range of applications. The nitroxide-mediated polymerization (NMP) method is a technique of choice to control the length, the composition and the architecture of the grafted copolymers. Nevertheless, cellulose is difficult to solubilize in organic media because of inter- and intramolecular hydrogen bonds. One possibility to circumvent this limitation is to solubilize cellulose in *N*,*N*-dimethylformamide (DMF) or *N*,*N*-dimethylacetamide (DMA) with 5 to 10 wt % of lithium salts (LiCl or LiBr), and carry out grafted polymerization in this medium. The stability of nitroxides such as SG1 has not been studied under these conditions yet, even though these parameters are of crucial importance to perform the graft modification of polysaccharide by NMP. The aim of this work is to offer a model study of the stability of the SG1 nitroxide in organic media in the presence of unprotected glucose or cellobiose (used as a model of cellulose) and in the presence of lithium salts (LiBr or LiCl) in DMF or DMA.

Contrary to TEMPO, SG1 proved to be stable in the presence of unprotected sugar, even with an excess of 100 molar equivalents of glucose. On the other hand, lithium salts in DMF or DMA clearly degrade SG1 nitroxide as proven by electron-spin resonance measurements. The instability of SG1 in these lithium-containing solvents may be explained by the acidification of the medium by the hydrolysis of DMA in the presence of LiCl. This, in turn, enables the disproportionation of the SG1 nitroxide into an unstable hydroxylamine and an oxoammonium ion.

Once the conditions to perform an SG1-based nitroxide-mediated graft polymerization from cellobiose have been established, the next stage of this work will be the modification of cellulose and cellulose derivatives by NMP.

## Introduction

With the rising costs and prospective shortage of fossil fuels, an increasing interest is dedicated to the elaboration of materials derived from renewable resources and in particular from natural polysaccharides [1–2]. However, one of the main drawbacks of polysaccharides is their inherently poor mechanical properties and heat resistance. To circumvent these limitations, one solution is the modification of polysaccharides by grafting synthetic polymers [3]. Three main strategies are generally reported to graft polymer chains onto polysaccharides, namely (i) the “grafting to” approach based on the end-functionalization of a preformed synthetic polymer chain grafted to the polysaccharide backbone, (ii) the “grafting through” method, which consists of copolymerizing premade vinyl-functionalized polysaccharides with a comonomer and (iii) the “grafting from” strategy where the grafted polymer chains grow from the polysaccharide backbone. This last method is the most convenient one as it is less sensitive to steric hindrance problems, which can limit the grafting process. The development of controlled/living radical polymerization (CLRP) techniques, such as atom transfer radical polymerization (ATRP) [4–7], reversible addition-fragmentation chain transfer (RAFT) [8–10], and nitroxide-mediated polymerization (NMP) [11], has opened new prospects in this research field, and permits precise tailoring of the synthetic chain length, the composition and the architecture [12].

**Figure 1:**
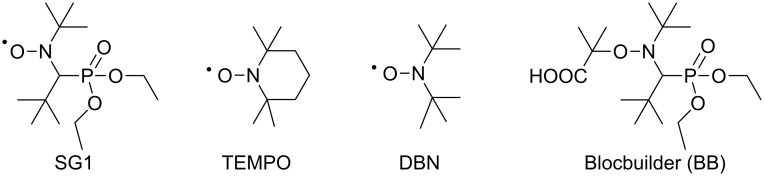
Structure of the SG1, TEMPO and DBN nitroxides and the BlocBuilder MA alkoxyamine.

Contrary to pullulan, dextran and locust bean gum, cellulose is not soluble in water and hardly soluble in few organic media. Consequently, its modification by polymer grafting proceeds either in organic media under homogeneous conditions or under heterogeneous conditions (surface-initiated polymerization from cellulose fibre, pulp, nanocrystals, etc. ) [2,13]. Working under homogeneous conditions allows for following the kinetics of the grafting copolymerization by size-exclusion chromatography (SEC) or NMR for instance. This is of crucial importance to prove the living/controlled character of a polymerization performed by a CLRP technique. A strategy to modify cellulose by polymer grafting under homogeneous conditions consists in previously modified cellulose into organosoluble cellulose-derivatives such as cellulose acetate, methyl, ethyl or hydroxypropylcellulose. The main drawback of this method of cellulose solubilisation is the loss of the initial polysaccharide, namely cellulose. Another strategy that has drawn interest in the scientific community is the use of ionic liquids [14]. However, these solvents usually are very expensive or must be specifically synthesized. A more convenient media, which has proven to be efficient to solubilize polysaccharides, is DMA in the presence of LiCl (generally 5 to 10 wt % versus DMA) [15]. This system has already been reported to successfully solubilize cellulose before grafting modification by ATRP in dimethyl sulfoxide (DMSO) [16], but not in the case of NMP yet. Indeed, to our knowledge, only hydroxyisopropylcellulose (HPC) has been modified by NMP under homogeneous conditions with the 2,2,6,6-tetramethyl-1-piperidinyloxy radical (TEMPO) (Figure 1) as a control agent and by using Barton ester intermediates [17]. Several hydroxyisopropylcellulose-grafted polystyrene (HPC-g-PS) were reported and the grafted PS chains, analysed by SEC after acid hydrolysis of the cellulose backbone, proved to be efficient to control the polymerization (number-average molar mass *M*_n_ ranging from 28,000 to 62,000 g·mol^−1^ and polydispersity index PDI ranging from 1.28 to 1.52). To our knowledge, the modification of free cellulose, i.e., cellulose with unprotected hydroxy groups, by NMP graft polymerization has not been studied yet. It has to be noted that sugar and in particular glucose have been used by Georges and co-workers [18] to degrade the TEMPO nitroxide to enhance the kinetics of styrene polymerization mediated by this nitroxide. In the case of a polysaccharide, this degradation reaction could be detrimental to the success of the grafted polymerization. In particular, the stability of the acyclic β**-**phosphorylated nitroxide *N*-(2-methylpropyl)-*N*-(1-diethylphosphono-2,2-dimethylpropyl)-*N*-oxyl) named SG1 by Tordo et al. [19–20], which is now recognized as the most potent nitroxide for NMP, in the presence of sugar and lithium-containing solvents has never been reported (Figure 1). It is noteworthy that the SG1 stability is a crucial parameter to ensure the success of the nitroxide-mediated polymerization.

The aim of this work is to propose a model study of the modification of unprotected cellulose by SG1-based nitroxide-mediated graft polymerization. In this study, glucose and cellobiose were used as a model of cellulose. The stability of the SG1 nitroxide was studied in the presence of unprotected glucose and lithium salts (LiBr and LiCl) in DMF or DMA by electron spin resonance (ESR). NMP of styrene was then performed with two SG1-based alkoxyamines (BlocBuilder MA alkoxyamine (BB)) and a cellobiose-BB alkoxyamine (called cello-SG1) with or without lithium salts. This study clearly proves that SG1 is stable in the presence of unprotected hydroxy functions, but that lithium salts in DMF or DMA degrade SG1. Two degradation mechanisms have been investigated, namely (i) redox reactions involving the SG1, (ii) the acid-catalyzed disproportionation of SG1 nitroxide into an unstable hydroxylamine and an oxoammonium ion.

## Results and Discussion

### Stability of SG1 in presence of glucose

**Figure 2:**
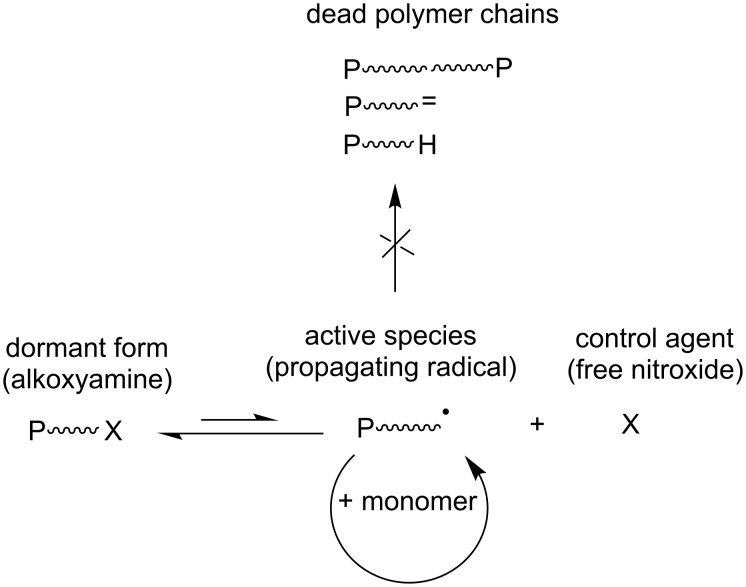
Key equilibrium between active and dormant species involved in the nitroxide-mediated (NMP) polymerization technique.

The NMP is based on a dynamic equilibrium between a propagating radical (P^•^) and a dormant form unable to propagate (alkoxyamine, i.e., radical end-capped by a nitroxide) in the presence of a nitroxide, which acts as a control agent (Figure 2). This reversible termination decreases the propagating radical concentration in the media and consequently the occurrence of irreversible terminations. Once the equilibrium between the dormant and active species is established, namely, the activation–deactivation equilibrium, all the chains grow at the same rate affording a living/controlled polymerization.

Several nitroxides have been synthesized since their first development in the 1980s [11]. In particular, the cyclic nitroxide TEMPO has been intensely studied [21–22] in the polymerization of styrene derivatives. Another important property of TEMPO is that it is reduced by reducing sugars such as glucose [18]. The consumption of nitroxides by reducing agents such as a sugar or a polysaccharide is of prime importance when aiming at the synthesis of glycopolymers or the graft polymerization onto polysaccharides. To our knowledge, Fukuda et al. were the only ones to polymerize by NMP an unprotected glycomonomer [23]. *N*-*(p*-Vinylbenzyl)-[*O*-β-D-galactopyranosyl-(1→4)]-D-gluconamide (VLA), a styrene derivative with an oligosaccharide moiety, was polymerized in DMF solution at 90 °C with di-*tert*-butyl nitroxide (DBN) as a control agent (Figure 1). The acetylation of VLA enabled the synthesis of well-defined glycopolymers with molar mass ranging from 2,000 to 40,000 g·mol^−1^ along with PDI values of about 1.1, whereas the NMP of VLA could not exceed *M*_n_ higher than 6,000, with PDI values increasing with conversion. According to the authors, chain transfer to the hydroxy groups of VLA was responsible for this phenomenon and lead to dead polymer chains and broad PDI values.

**Figure 3:**
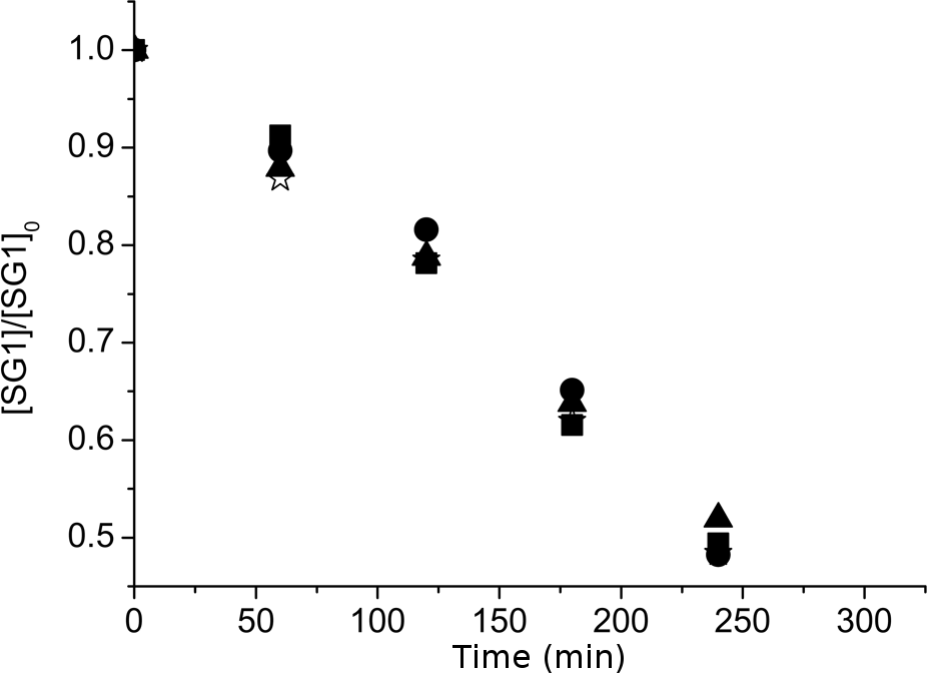
Degradation of the SG1 nitroxide versus time in the presence of 0 (empty stars), 1 (filled squares), 10 (filled triangles) and 100 (filled circles) mol equivalents of D-glucose as measured by ESR in *t*-BuPh at 25 °C (samples heated in DMA at 120 °C and then diluted in *t*-BuPh).

**Figure 4:**
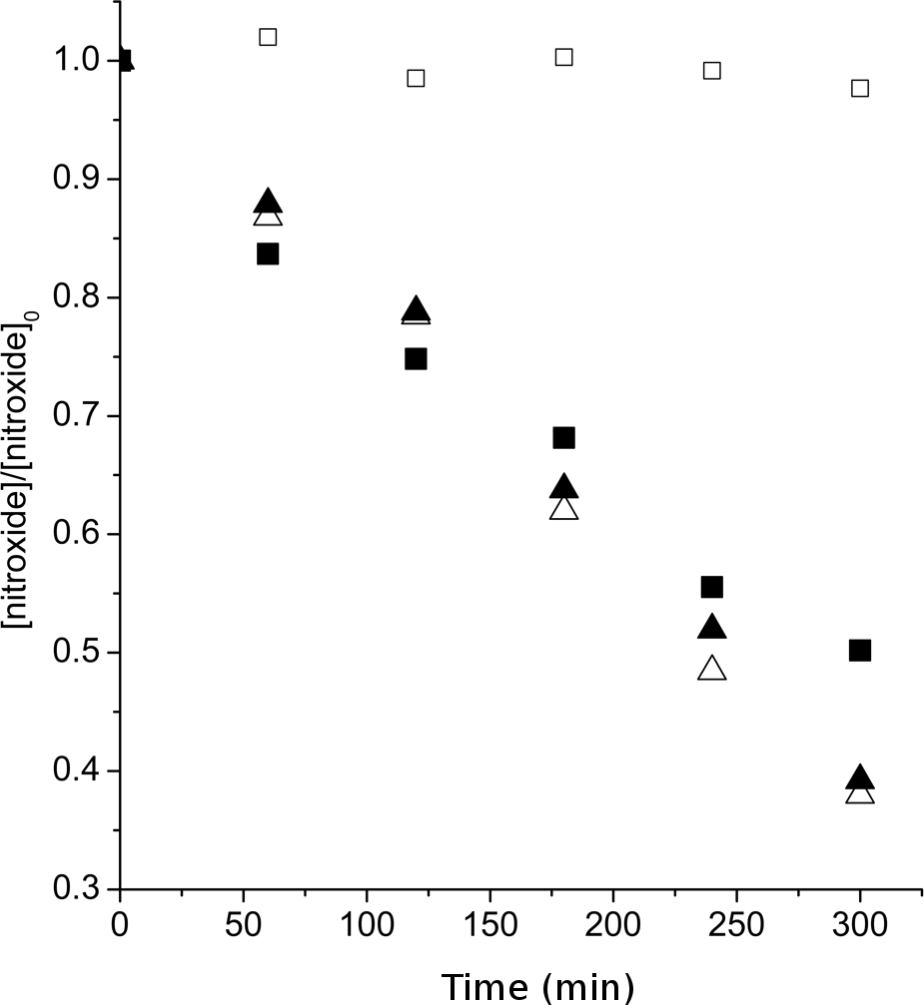
Degradation of the SG1 (triangles) and TEMPO (squares) nitroxides versus time in the presence of 0 (empty symbols) and 10 (filled symbols) mol equivalents of D-glucose as measured by ESR in *t*-BuPh at 25 °C (samples heated in DMA at 120 °C and then diluted in *t*-BuPh).

The first part of this work was devoted to studying the stability of SG1 in the presence of D-glucose. SG1 (1.5 × 10^−2^ mol·L^−1^) in DMA was heated at 120 °C for 5 hours in the presence of different amounts of D-glucose. Samples were regularly withdrawn from the media and diluted in *tert*-butylbenzene (*t*-BuPh) before analysis by ESR. Irrespective of the number of molar equivalents of glucose (from 1 to 100 versus SG1), an increase of the SG1 consumption was not observed when compared to the experiment without glucose (Figure 3). In contrast, the concentration of TEMPO decreased by 50% after 5 hours in the presence of 10 mol equivalents of D-glucose (Figure 4). These ESR results prove the lack of interaction of the SG1 with unprotected sugars leading to extra degradation of the nitroxide. Furthermore, these results are very encouraging to envision the modification of unprotected polysaccharides by grafting synthetic polymer chains by the SG1-based NMP technique.

### Stability of SG1 in the presence of lithium salts

The system DMA/LiCl has been largely reported for the analysis of cellulose and for the preparation of a wide variety of its derivatives [15]. This system is of particular interest for analysis as it is colourless and efficient to dissolve polysaccharides without or at least with negligible degradation even in the case of high molar mass. Even though it is not proven yet, replacing the DMA molecules as complexing agents around the Li^+^ ions by cellulose-hydroxy groups is the proposed mechanism to explain the cellulose dissolution [14].

**Figure 5:**
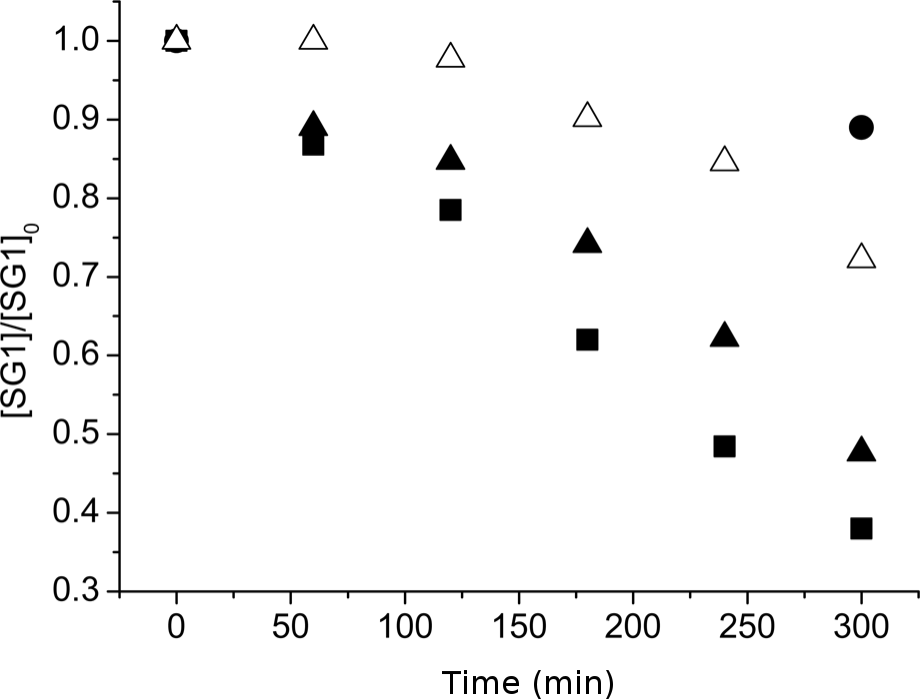
Degradation of the SG1 nitroxide versus time in DMA (filled squares), DMF (filled triangles), MeOH (filled circles) and *t*-BuPh (empty triangles) as measured by ESR in *t*-BuPh at 25 °C (samples heated in DMA, DMF, MeOH or *t*-BuPh at 120 °C, removed after 1 to 5 hours and diluted in *t*-BuPh).

Although many nitroxide-mediated polymerizations were performed in pure DMA or DMF [24–27], such polymerizations have never been performed in DMF or DMA in the presence of LiCl or LiBr. In particular, the stability of SG1 nitroxide has never been studied in DMA or DMF in the presence of LiCl or LiBr. The stability of the SG1 nitroxide in various solvents (DMA, DMF, MeOH and *t*-BuPh) without lithium salt was first investigated by ESR (Figure 5). In accordance with previous experiments, SG1 solutions in DMA, DMF, MeOH or *t*-BuPh (1.5 × 10^−2^ mol·L^−1^) were heated at 120 °C and then diluted in *t*-BuPh for ESR analysis at 25 °C. The SG1 degradation is clearly faster in DMA or DMF compared to *t*-BuPh and MeOH. After 5 hours of heating at 120 °C, only 10 and 25% of SG1 were consumed in MeOH and *t*-BuPh, respectively, whereas 50 and 60% of SG1 degraded in DMF and DMA, respectively. SG1 is therefore less stable in DMF or DMA than in MeOH or *t*-BuPh.

**Figure 6:**
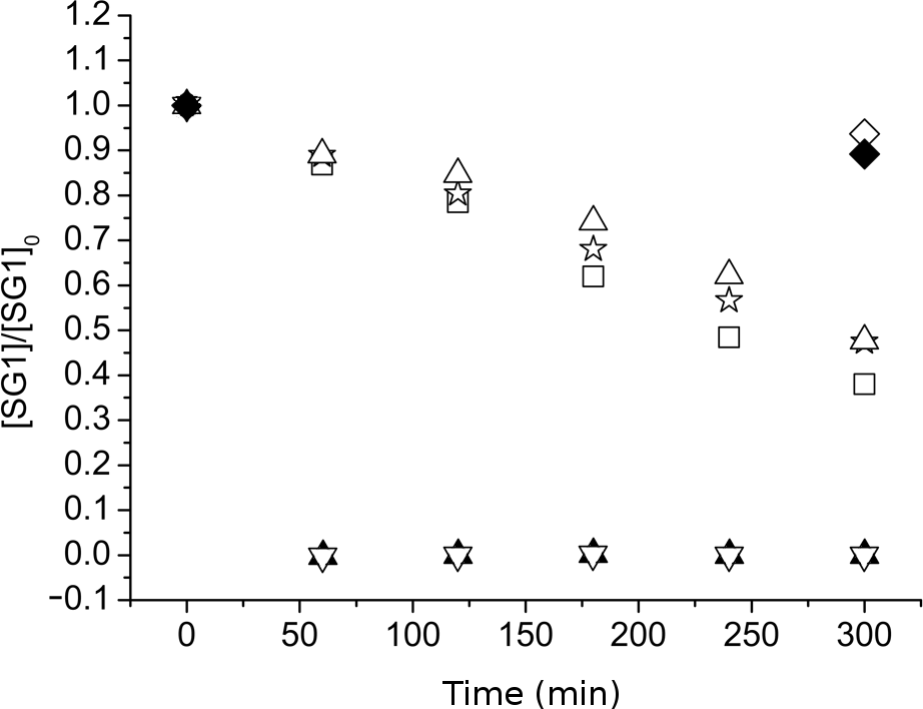
Degradation of the SG1 nitroxide versus time in the presence of 0% of lithium salt in DMA (empty squares); DMF (empty triangles) and MeOH (empty diamonds), 4.5 or 10 wt % of LiCl in DMA or DMF (filled triangles), 4.5 wt % of LiBr in DMA (reverse filled triangles), 4.5 wt % of LiCl in MeOH (filled diamonds) and 4.5 wt % of NaCl in DMA (empty stars) as measured by ESR in t-BuPh at 25 °C (samples heated in DMA, DMF or MeOH at 120 °C, removed after 1 to 5 hours and diluted in t-BuPh).

The influence of the lithium salt concentration (LiCl or LiBr) was then investigated at 4.5 and 10 wt % in DMF and DMA (Figure 6).

**Figure 7:**
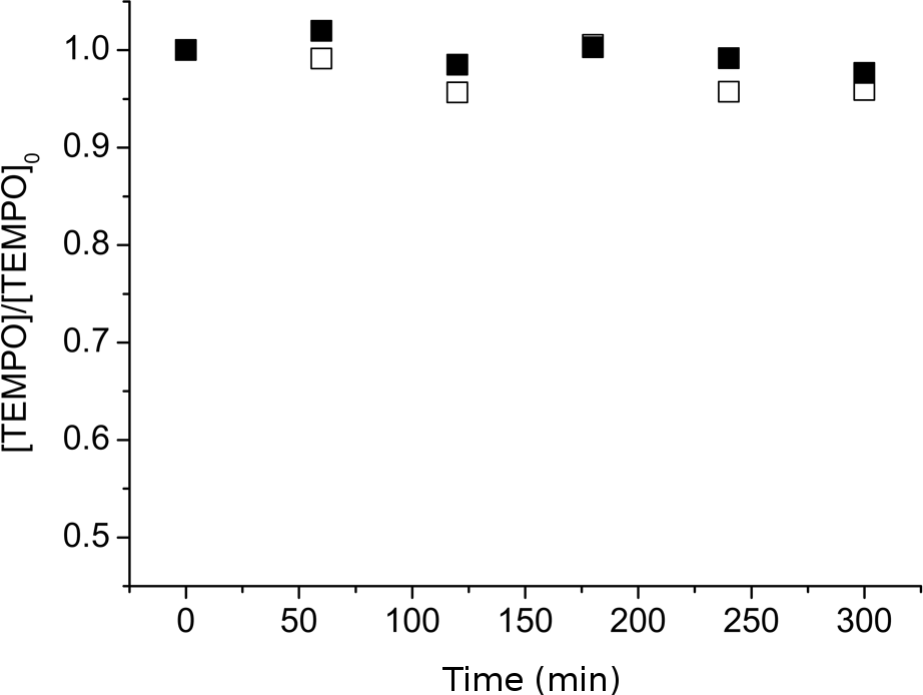
Degradation of the TEMPO nitroxide versus time in DMA in the presence of 0% lithium salt (empty squares) and 4.5 wt % LiCl (filled squares) as measured by ESR in *t*-BuPh at 25 °C (samples heated at 120 °C in DMA, removed after 1 to 5 hours and diluted in *t*-BuPh).

Figure 6 clearly proves that irrespective of the counter ion (Cl^−^ or Br^−^), SG1 was totally consumed within 1 hour with 4.5 or 10 wt % of lithium salt in DMF or DMA. In contrast to LiCl and LiBr, NaCl did not seem to catalyse the SG1 degradation. It is noteworthy that this experiment was performed under heterogeneous conditions since NaCl is not soluble in DMA. Although LiCl or LiBr degrades the SG1 nitroxide in DMF or DMA, degradation does not occur when these lithium salts are dissolved in methanol (Figure 6). The combination of the solvent (DMF or DMA) with the lithium salt (LiCl or LiBr) is, thus, responsible for the SG1 degradation and not the lithium salt alone. Two possible SG1 degradation mechanisms will be discussed in the last part of this work. Nevertheless, one notices that contrary to SG1, the TEMPO nitroxide proved to be stable in DMA even with 4.5 wt % of LiCl (Figure 7). The instability of SG1 in lithium-containing solvents such as DMF, DMA/LiCl or LiBr can consequently not be extended to all nitroxides.

**Figure 8:**
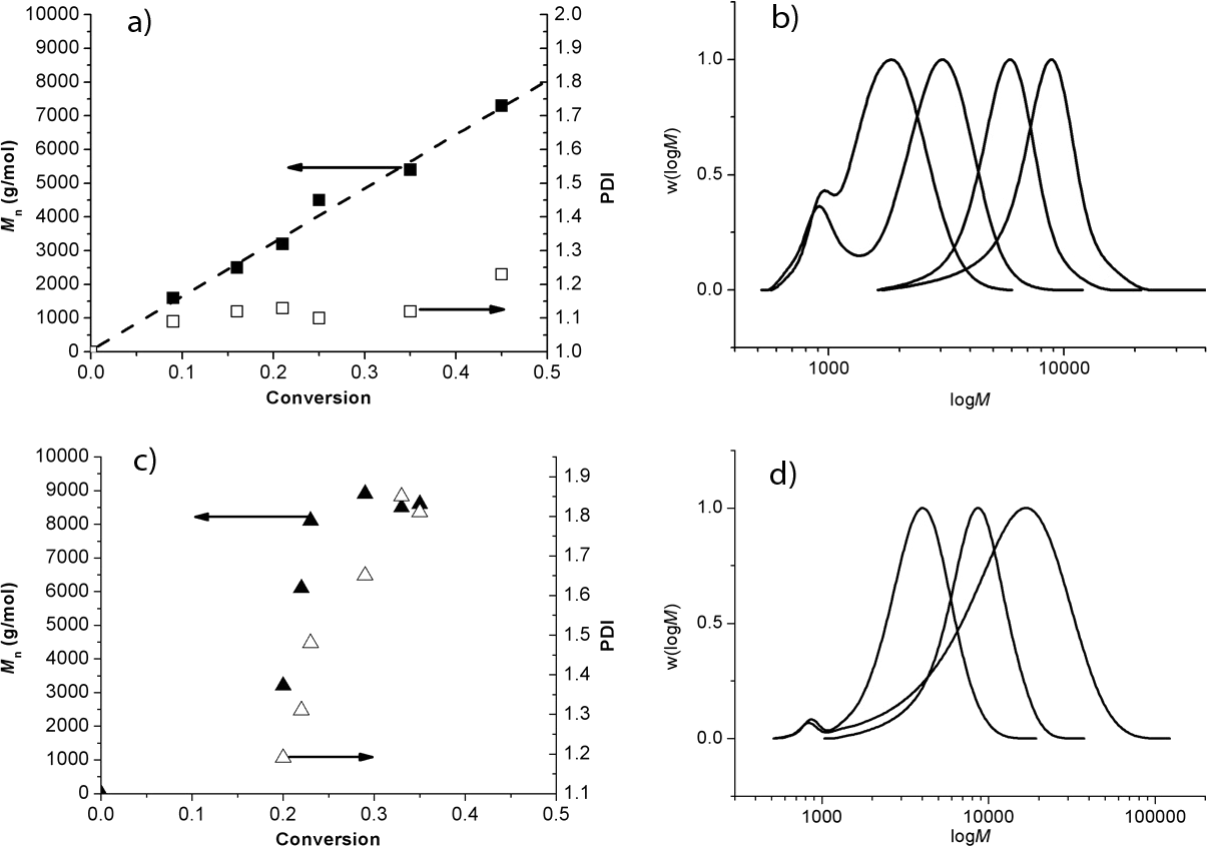
NMP of styrene initiated by the BlocBuilder MA alkoxyamine at 120 °C in DMA without LiCl salt. (a) Evolution of the number-average molar mass (*M*_n_: filled symbols, linear fit: dash lines) and polydispersity indexes (PDI: empty symbols) with conversion. (b) Molar mass distribution (w(log*M*) versus log*M*) of the polystyrene chains at various conversions. NMP of styrene initiated by the BB alkoxyamine at 120 °C in DMA with 4.5 wt % of LiCl. (c) Evolution of the number-average molar mass (*M*_n_: filled symbols) and polydispersity indexes (PDI: empty symbols) with conversion. (d) Molar mass distribution (w(logM) versus logM) of the polystyrene chains at various conversions.

According to these ESR results, it is not a good option to carry out a controlled/living polymerization by SG1-based NMP in DMA/LiCl or DMF/LiCl. As expected, with the BlocBuilder MA alkoxyamine as an initiator (Figure 1), the NMP of styrene performed in DMA at 120 °C without LiCl fulfilled the criteria of a controlled polymerization (PDI values < 1.5, linear increase of *M*_n_ values with conversion (Figure 8a), and a regular shift of the molar mass distribution towards high *M*_n_ (Figure 8b)). In contrast, the control of the polymerization was lost under the same conditions after 1.5 hours in the presence of 4.5 wt % of LiCl (PDI > 1.5, *M*_n_ values constant around 8,000 g·mol^−1^ (Figure 8c), and a broadening of the molar mass distribution (Figure 8d)).

**Scheme 1:**
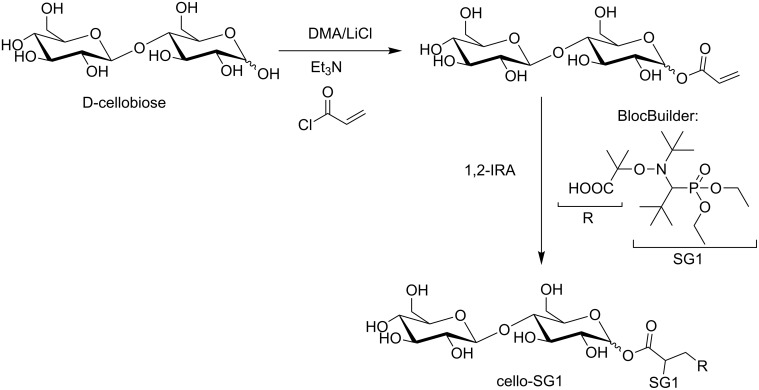
Synthesis of the cellobiose and SG1-based alkoxyamine (cello-SG1). The shown regioisomer exhibits an acroylation in C1 since this OH group is known to be more reactive, but all regioisomers are possible.

To mimic a cellulose chain functionalized by a SG1-based alkoxyamine, we coupled the commercially available BlocBuilder MA alkoxyamine and cellobiose to prepare a compound called cello-SG1 (Scheme 1). As the aim was to prepare a model of cellulose, we targeted the grafting of one BB unit onto cellobiose but without selecting one specific position. Indeed, in the case of cellulose polysaccharide, it is very challenging to selectively modify only one type of hydroxy function (C_2_-OH, C_3_-OH or C_6_-OH).

**Figure 9:**
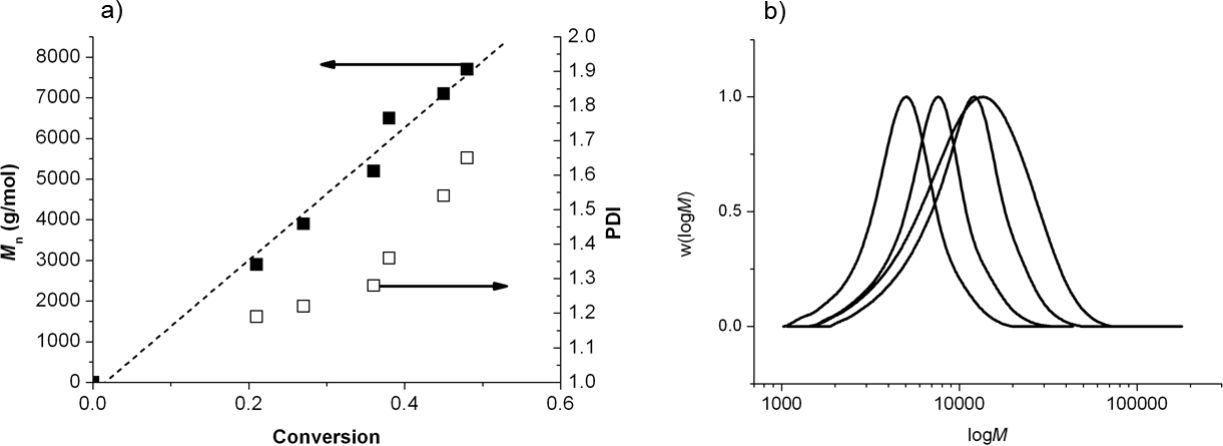
(a) Evolution of the number-average molar mass (*M*_n_: filled symbol, linear fit: dash lines) and polydispersity index (PDI: empty symbols) with styrene conversion by using cello-SG1 as initiator. (b) Molar mass distribution (w(log*M*) versus log*M*) of the polystyrene chains at various conversions (DMA, 120 °C, [cello-SG1]_0_ = 1.5 × 10^−2^ mol·L^−1^, [styrene]_0_ = 1.4 mol·L^−1^).

The procedure of elaboration of this alkoxyamine has already been described elsewhere [28]. Briefly, the synthesis of the cello-SG1 alkoxyamine is performed in two steps: (i) acroylation of one hydroxy function of cellobiose to graft one acrylate function, (ii) intermolecular 1,2-radical addition (1,2-IRA) of the SG1-based BlocBuilder MA alkoxyamine onto the acrylate function. Nevertheless, in the case of cellobiose the first step of acroylation has to be performed in DMA with a minimal amount of LiCl to ensure the cellobiose solubilization. The cello-SG1 alkoxyamine was then used to initiate the NMP of styrene in DMA at 120 °C. Figure 9a and Figure 9b prove that a successful controlled/living polymerization could be achieved in these conditions (linear increase of the number-average molar mass with conversion, PDI values < 1.5, and regular shift of the molar mass distribution) until 40% of conversion. For higher conversion values, the PDI values increase (>1.5), and the molar mass distribution broadens because of SG1 degradation due to residual lithium salt in the media that is very difficult to fully eliminate after the 1,2-IRA step.

This study therefore clearly proves that lithium salt can be used to solubilize sugar, oligo- or polysaccharides, but that they have to be carefully removed before performing a SG1-based NMP. In addition, the successful NMP initiated by the cellobiose-derivative alkoxyamine is a very promising result to envision the modification of fully or partially unprotected polysaccharide by SG1-based nitroxide-mediated graft (co)polymerization.

### Mechanism of the degradation of the SG1 nitroxide in DMF or DMA/LiCl or LiBr solvent

ESR experiments proved that the SG1 nitroxide was degraded in DMF or DMA with 4.5 wt % of LiCl or LiBr. Two hypotheses could be postulated to explain this degradation: (i) the SG1 undergoes an oxidation reaction, (ii) LiCl catalyses the hydrolysis of DMA (or DMF) that degrades the SG1 into unstable species.

#### Oxidation of the SG1 nitroxide

**Scheme 2:**
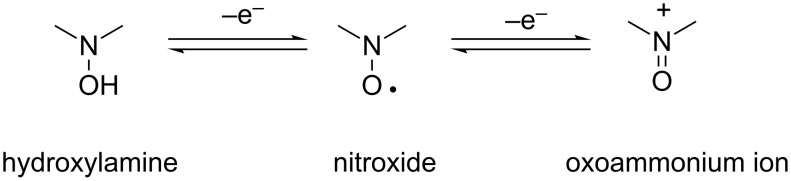
(Reversible) redox system of nitroxide.

Nitroxide can be readily oxidized into an oxoammonium ion and reduced to hydroxylamine (Scheme 2) [29]. This feature was extensively used by many researchers for developing a pure organic battery based on the nitroxide functionality [30]. The redox potentials of cyclic nitroxides and in particular TEMPO are well-known in acetonitrile, but there was only one measurement for SG1 in acetonitrile and no measurement in DMA or DMF as a solvent. The aim of the study was to measure the redox potentials of the SG1 and LiCl/solvent and to conclude if a redox reaction could happen between these species.

**Figure 10:**
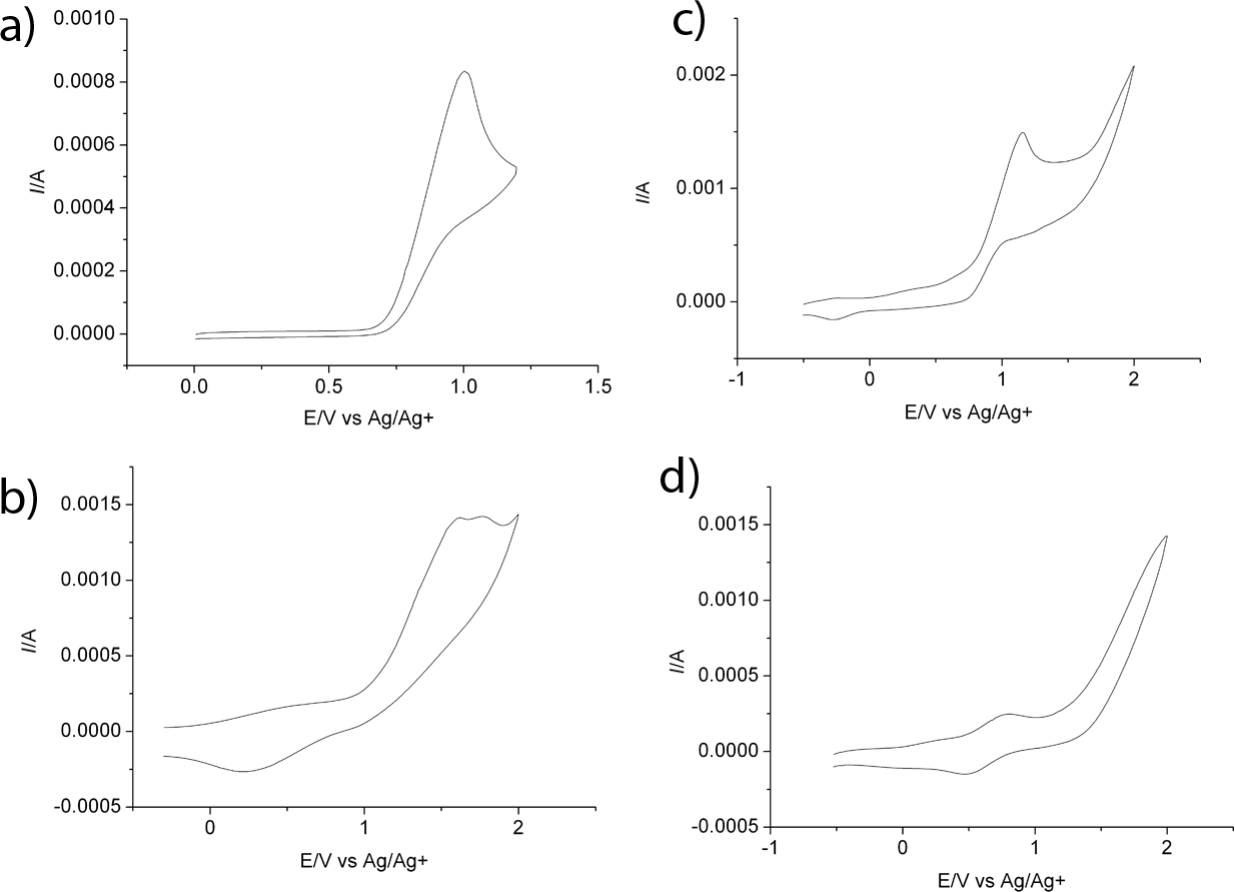
Cyclic voltammograms in DMF of (a) SG1 (10^−2^ M)/ NaClO_4_ (10^−1^ M) and (b) LiCl (10^−2^ M)/TBAPF_6_ (10^−1^ M). Cyclic voltammograms in MeOH of (c) SG1 (10^−2^ M)/TBAPF_6_ (10^−1^ M) and (d) LiCl (10^−2^ M)/TBAPF_6_ (10^−1^ M). Glassy carbon electrode, Ag/AgCl, Pt, sweep rate 50 mV·s^−1^.

The redox potentials of SG1 and LiCl (4.5 wt %) in DMF and MeOH were then determined by cyclic voltammetry with a platinum electrode and are listed in Table 1. The cyclic voltammograms of SG1 and LiCl in DMF and MeOH are presented in Figure 10. The redox potential of the reduction of Li^+^/Li in DMF is around −2.1 V as already reported [31].

**Table 1:** Experimental redox potential of the oxidation (*E*_ox_) and of the reduction (*E*_red_) in DMF and MeOH.^a^

	*E*_ox_ or *E*_red_ (vs Ag/AgCl)

SG1 (DMF)	*E*_ox_ = 0.64 *E*_red_ = 0.00
Cl_2_/Cl^−^ (DMF)	*E*_ox_ / *E*_red_ = 0.86
SG1 (MeOH)	*E*_ox_ = 0.64 *E*_red_ = 0.00
Cl_2_/Cl^−^ (MeOH)	*E*_ox_ = 1.10 *E*_red_ = 0.84

^a^Glassy carbon electrode, Ag/AgCl, Pt, sweep rate 50 mV·s^−1^.

**Figure 11:**
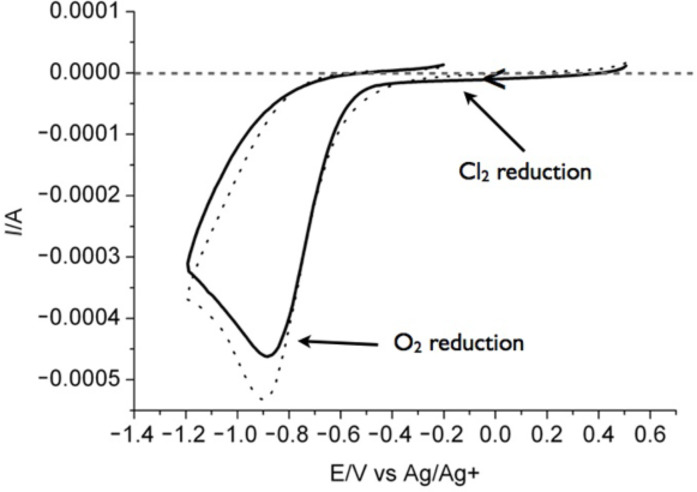
Cyclic voltammograms of DMF/4.5 wt % LiBr solution heated at 80 °C for 30 min (plain line) and 14 hours (dotted line). Glassy carbon electrode, Ag/AgCl, Pt, sweep rate 50 mV·s^−1^.

Initially only the SG1 nitroxide, Li^+^ and Cl^−^ were present in the media. The only possible redox reaction is the one involving the reduction of the in situ formed Cl_2_ to Cl^−^ and the oxidation of the nitroxide to an oxoammonium ion. In conclusion, if Cl_2_ can be formed in the system, then the oxidation of the SG1 nitroxide is possible. However, this hypothesis is challenging to validate since Cl_2_ is a gas and thus difficult to quantify. We tried to prove the formation of Br_2_ by heating DMF/4.5 wt % LiBr solutions for 14 hours. A comparison with the same solution but with heating for only 30 min did not show any difference by cyclic voltammetry. In particular, the reduction wave of Br_2_ into Br^−^ did not increase with prolonged heating time (Figure 11). Since the formation of Cl_2_ or Br_2_ in the case of DMA/LiCl or DMA/LiBr was not straightforward, the hypothesis of the SG1 degradation by a redox phenomenon was hardly acceptable. A different explanation was then investigated, namely the disproportionation of the SG1 nitroxide into unstable species in acidic media.

#### Disproportionation of SG1 nitroxide into unstable species in acidic media

**Scheme 3:**
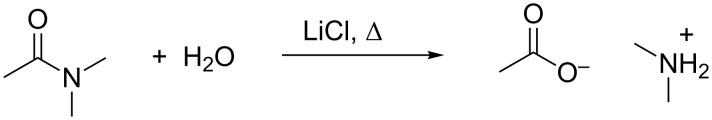
Hydrolysis of DMA in the presence of LiCl.

LiCl has proven to catalyze the hydrolysis of DMA [32]. In particular, heating wet DMA in the presence of LiCl produces dimethylammonium acetate and its conjugated base dimethylamine (Scheme 3). The DMA/LiCl solution becomes more acidic with time as the dimethylamine is volatile and gradually evaporates, and the acetic acid accumulates (pH decreases from 7 to 4–5). The increase of the acidity accelerates the DMA hydrolysis since the reaction mechanism between water and DMA becomes an acid-catalyzed process.

**Scheme 4:**
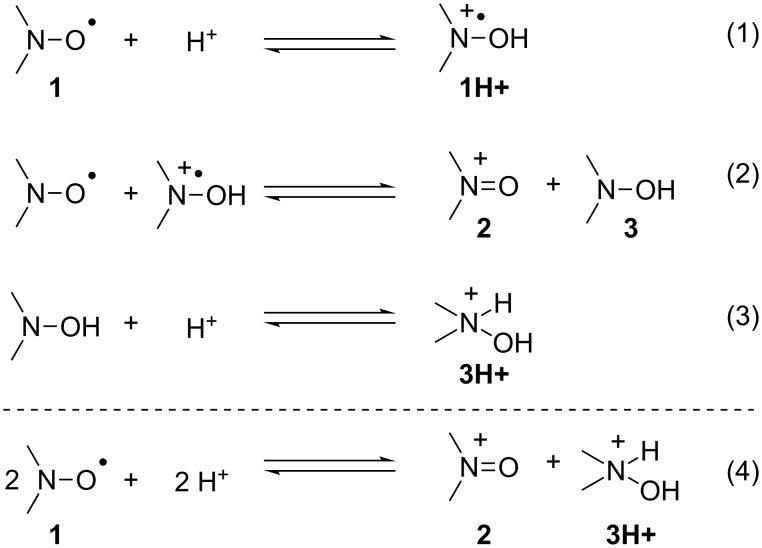
Disproportionation of nitroxide **1** by acid treatment.

The disproportionation of TEMPO nitroxide in acidic media is a well-documented degradation process [33–34]. In particular, in the presence of an excess of acid HX (X = BF_4_, Cl^−^, Br^−^, Br_3_^−^, OCl_4_^−^), the disproportionation of TEMPO gives rise to the oxoammonium salt along with the protonated hydroxylamine (Scheme 4).

Concerning the hydrolysis of DMA and the disproportionation of TEMPO in acidic media, we could postulate that the hydrolysis of DMA in the presence of LiCl forms HCl, which in turn, degrades SG1 into oxoammonium salt and hydroxylamine. However, Bagryanskaya et al. [35] proved that the hydroxylamine derivative from SG1 is unstable. The cyclic voltammogram of SG1 also proved the instability of the SG1 oxoammonium cation since its reversible reduction is not observed (Figure 10). In conclusion, the degradation of SG1 in DMA/LiCl solutions could be explained by a disproportionation mechanism in the presence of HCl giving rise to an unstable hydroxylamine and an oxoammonium cation. It is noteworthy that the instability of SG1 has already been observed at 75 °C at pH 3.5 in the presence of methacrylic acid [36].

**Figure 12:**
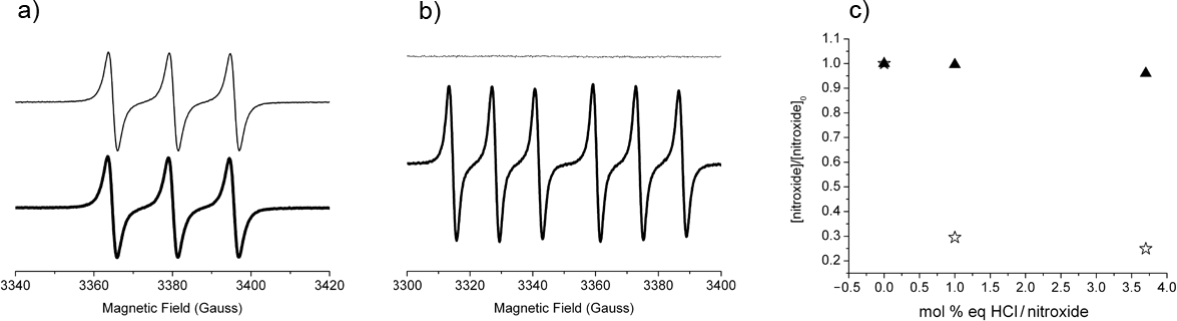
Degradation of the nitroxides (SG1 and TEMPO) in the presence of HCl. (a) TEMPO ESR signal in the presence of 3.7 mol % equivalents of HCl at *t* = 0 (thick line) and *t* = 5 h (thin line). (b) SG1 ESR signal in the presence of 3.7 mol % equivalents of HCl at *t* = 0 (thick line) and *t* = 5 h (thin line). c) SG1 (filled triangles) and TEMPO (empty stars) initial degradation (*t* = 0) in the presence of 0, 1 and 3.7 mol % equivalent of HCl versus nitroxide (ESR in *t*-BuPh at 25 °C, samples heated at 120 °C in MeOH, and diluted in *t*-BuPh).

To confirm the instability of the SG1 in acidic media and to explain the difference between SG1 and TEMPO when heated in DMA/LiCl (see ESR experiments and Figure 6 and Figure 7), SG1 and TEMPO MeOH solutions (1.5 × 10^−2^ mol·L^−1^) were heated at 120 °C in the presence of HCl (3.7 mol % equivalents). Samples were withdrawn regularly and diluted in *t*-BuPh for ESR analysis (Figure 12). In accordance with previous observations in DMA/LiCl, the kinetics of the consumption of SG1 and TEMPO in the presence of HCl are very different. TEMPO degrades very quickly in the first seconds of the experiment (Figure 12c) to reach an equilibrium (TEMPO concentration constant with time for 5 h, Figure 12a), whereas the SG1 consumption is slower (Figure 12c) but complete since all the nitroxide is consumed after 5 h at 120 °C (Figure 12b). This experiment proves that the acidity of the media (DMA/LiCl) is responsible for the SG1 degradation. The difference between TEMPO and SG1 nitroxides could be explained by a disproportionation mechanism that quickly gives a stable hydroxylamine and an oxoammonium ion in the case of TEMPO and slowly gives the same but unstable species in the case of SG1. The equilibrium (1) and (3) (Scheme 4) are then shifted towards (**1H+**) and (**3H+**) formation up to the complete consumption of the SG1 nitroxide.

## Conclusion

This study proved that contrary to TEMPO nitroxide, SG1 was stable in the presence of unprotected glucose. More specifically, SG1 was stable in the presence of up to 100 mol equivalents of glucose when heated at 120 °C in DMA. In contrast, SG1 degraded in DMF or DMA in the presence of LiCl or LiBr lithium salts, whereas TEMPO remained stable. ESR analyses proved that the lithium salt itself was not responsible for the nitroxide degradation, but that the degradation was caused by the combination of the solvent (DMF or DMA) with the lithium salt (LiCl or LiBr).

The first hypothesis to explain the SG1 consumption in DMA/LiCl was based on the SG1 oxidation by Cl_2_ (or Br_2_ in the case of LiBr lithium salt). However, the study by cyclic voltammetry of the redox potential of SG1 and LiCl could not attest to this hypothesis, since the presence of Cl_2_ (or Br_2_) in the media could not be proven.

Ultimately, the instability of SG1 in DMA/LiCl was attributed to the disproportionation of the nitroxide in acidic media forming hydroxylamine and an oxoammonium ion, which were stable in the case of TEMPO and unstable in the case of SG1.

The successful nitroxide-mediated polymerization of styrene in DMA from an alkoxyamine based on cellobiose and SG1 confirmed the ability to perform SG1-based NMP in DMA (without lithium salts) in the presence of unprotected sugars, and consequently opened the way to the modification of cellulose polymer chains by NMP.

## Experimental

### Materials and general experimental details

Triethylamine (Et_3_N, Aldrich, 99+%), acryloyl chloride (Aldrich, 97+%), D*-*glucose (Aldrich, 99.5%), D*-*cellobiose (Alfa Aesar, 98+%), lithium chloride (LiCl, Aldrich, 99+%), lithium bromide (LiBr, Aldrich, 99+%), sodium perchlorate (NaClO_4_, Aldrich, ACS reagent ≥98%), tetrabutylammonium hexafluorophosphate (TBAPF_6_, Fluka, 98+%), TEMPO (Aldrich, 98%), styrene (Aldrich, 99+%), *N*,*N*-dimethylformamide (DMF, SDS, Analytical grade), *N*,*N*-dimethylacetamide (DMA, Aldrich, 99.5+%), sodium chloride (NaCl, Carlo Erba), ethyl acetate (EtOAc, Carlo Erba), triethyl phosphite (P(O)(OEt)_3_, Aldrich, 99+% ), and *tert*-butylbenzene (*t*-BuPh, Aldrich, 99%) were used as received. The 2-methyl-2-(*N*-*tert*-butyl-*N*-(1-diethoxyphosphoryl-2,2-dimethylpropyl)aminoxy)-propionic acid alkoxyamine (BlocBuilder MA, BB) was kindly supplied by Arkema.

The styrene conversion was estimated by ^1^H NMR experiments on a Bruker Avance 400 spectrometer (DMSO-*d*_6_ as solvent, 400 MHz; chemical shifts are given relative to TMS used as an internal reference). ^31^P and ^1^H NMR analyses were performed on a Bruker Avance 400 spectrometer in DMSO-*d*_6_.

The size-exclusion chromatography analyses were performed by using an EcoSEC system from TOSOH equipped with a differential refractometer detector. THF was used as an eluent with 0.25 vol % toluene as a flow marker at a flow rate of 0.3 mL·min^−1^ after filtration on Alltech PTFE membranes with a porosity of 0.2 µm. The column oven was kept at 40 °C, and the injection volume was 20 µL. One ResiPore Pre-column (50 mm, 4.6 mm) and two ResiPore columns (250 mm, 4.9 mm) from Polymer Laboratories were used in series. The system was calibrated by using polystyrene standards in the range 100–400,000 g·mol^−1^, purchased from Agilent.

Electron spin resonance experiments were performed on a Bruker EMX 300 spectrometer and *t*-BuPh was used as a solvent. SG1 (or TEMPO) solutions (1.5 × 10^−2^ mol·L^−1^) were deoxygenated under argon and then heated at 120 °C for 5 hours in Schlenk tubes. Samples (134 μL) were regularly withdrawn and diluted in 20 mL of *t*-BuPh for analysis at 25 °C under air.

The electrochemical experiments were conducted in a three-electrode glass cell. A glassy carbon disk electrode (1 cm^2^) and a platinum wire were used as the working and the counter electrodes, respectively. The potentials were referred to Ag/AgCl. The cyclic voltammograms were collected at room temperature and atmosphere at a potential sweep rate of 50 mV·s^−1^ generated by a potentiostat/galvanostat (Solartron analytical SI 1287). In all the experiments, the concentration of SG1 and LiCl was 10^−2^ mol·L^−1^ and that of the supporting electrolyte (NaCLO_4_ or TBAPF_6_) 0.1 mol·L^−1^.

High-resolution mass spectrometry experiments were performed by using a QStar Elite mass spectrometer (Applied Biosystems SCIEX, Concord, ON, Canada) equipped with an electrospray ionization source operated in the positive mode. The capillary voltage was set to +5,500 V and the cone voltage to +20 V. Air was used as the nebulizing gas (10 psi), while nitrogen was the curtain gas (20 psi). In this hybrid instrument, ions were measured by using an orthogonal acceleration time-of-flight (oa-TOF) mass analyser. Instrument control, data acquisition, and data processing were achieved by using Analyst software (QS 2.0) provided by Applied Biosystems. The sample solution (i.e., cello-SG1 diluted in methanol containing 3 mM of ammonium acetate) was introduced in the ionization source at a 5 μL min^−1^ flow rate by using a syringe pump.

### Synthesis of the cellobiose and SG1-based alkoxyamine (Cello-SG1)

D-cellobiose (10 g, 29 mmol) was dissolved in a solution of DMA/LiCl (94 mL/1.5 g) at 100 °C for 20 min under argon atmosphere. After cooling, Et_3_N (4.5 mL, 32 mmol) was added at room temperature, and acryloyl chloride (2.4 mL, 29 mmol) was introduced dropwise at 0 °C for 10 min (0.24 mL/min). The temperature was kept at 0 °C for 20 min. The mixture was then heated at 40 °C for 2 h. Triethylammonium chloride was removed by filtration at room temperature and the filtrate (98 mL) was kept for the next step. ESI^+^ (mass spectrometry) analyses proved the presence of a mixture of cellobiose mono, di- and tri-acroylated, acrylic acid (resulting from the hydrolysis of acryloyl chloride), and free cellobiose. It is noteworthy that this reaction is not regioselective, so that several regioisomers are formed, which are very difficult to separate and quantify. BB (4.8 g, 12.6 mmol) was added to the previous filtrate (50 mL). After deoxygenation by argon bubbling for 20 min at room temperature, the solution was heated at 80 °C for 3 h. After cooling, EtOAc was added to the media and the product was recovered by precipitation. The obtained white solid was dried under reduced pressure to give the cellobiose-based alkoxyamine cello-SG1 (two steps yield reaction: 13%, measured by ^31^P NMR with P(O)(OEt)_3_ as an internal reference).

The *E*_a_ value of the cello-SG1 homolysis was determined by ^31^P NMR as already described (*E*_a_ = 122 kJ·mol^−1^) [37–38].

The protonated molecule (C_32_H_61_NO_18_P^+^, *m*/*z*_theo_ 778.3621) was accurately mass measured with a relative error of ±0.3 ppm by using two reference ions from a poly(propylene glycol) as internal standards.

### Nitroxide-mediated polymerization of styrene in *N*,*N*-dimethylacetamide

After the addition of styrene (2.16 g, 20.7 mmol) to the alkoxyamine solution (BB or Cello-SG1, 0.23 mmol, previously dissolved in 15 mL of DMA), the solution was deoxygenated by argon bubbling for 20 min at room temperature and then heated at 120 °C for 5 h. Aliquots were periodically withdrawn during the reaction time and cooled in an iced water bath to quench the polymerization. For all of them, the monomer conversion was determined by ^1^H NMR analysis of the raw polymerization medium in DMSO-*d*_6_ solution, whereas the *M*_n_ and the PDI of the polystyrene chains were measured by SEC/THF.
